# 17β-Estradiol Inhibits PCSK9-Mediated LDLR Degradation Through GPER/PLC Activation in HepG2 Cells

**DOI:** 10.3389/fendo.2019.00930

**Published:** 2020-01-30

**Authors:** Wei Fu, Xiao-Ping Gao, Sheng Zhang, Yan-Ping Dai, Wen-Jun Zou, Li-Min Yue

**Affiliations:** ^1^Department of Physiology, West China School of Basic Medical and Forensic Medicine, Sichuan University, Chengdu, China; ^2^College of Medicine, Chengdu University of Traditional Chinese Medicine, Chengdu, China

**Keywords:** 17β-estradiol, GPER activation, PCSK9, LDLR degradation, clathrin

## Abstract

Plasma levels of PCSK9 are significantly higher in postmenopausal women. Pharmacologically increased estrogen levels have been shown to lower PCSK9 and LDL-C levels in animals and humans. The action of estrogen suggests that it has the ability to prevent PCSK9-mediated LDLR degradation in liver cells. However, little is known about how estrogen alters PCSK9-mediated LDLR degradation. Here, we report that 17β-estradiol (βE2) reduces PCSK9-mediated LDLR degradation by a mechanism that involves activation of the G protein-coupled estrogen receptor (GPER). In cultured HepG2 cells, βE2 prevented the internalization of PCSK9, which subsequently lead to PCSK9-mediated LDLR degradation. The altered LDLR levels also resulted in an increase in LDL uptake that was not observed in the absence of PCSK9. In addition, we showed that clathrin was rapidly increased in the presence of PCSK9, and this increase was blocked by βE2 incubation, suggesting rapid recruitment of clathrin in HepG2 cells. PLCγ activation and intracellular Ca^2+^ release were both increased due to the rapid effect of estrogen. By using a GPER antagonist G15, we demonstrated that the GPER mediates the action of estrogen. Together, the data from this *in vitro* study demonstrate that estrogen can regulate LDLR levels mainly through GPER activation, which prevents PCSK9-dependent LDLR degradation in HepG2 cells.

## Introduction

Estrogen plays a role in lipid metabolism in both physiological and pharmacological contexts. Because of the benefits of estrogen, as documented in the past decade, premenopausal women have experience delayed first-time myocardial infarction (1–3) and have approximately one half the risk of cardiovascular disease relative to that of men (4, 5). Estrogen replacement therapy is still controversial, and supplements with a high dose of estrogen plus progestin or estrogen alone have been associated with a high risk of developing breast cancer (6–9). A large randomized trial (10) from the Women's Health Initiative (WHI) indicates that estrogen plus progestin is not beneficial in postmenopausal women; there is early harm for coronary heart disease (CHD) as well as continuing harm for stroke and venous thromboembolic events (VTE). The WHI results are similar to those reported by others (11–13). However, estrogen administered to postmenopausal women increases LDL clearance and lowers the levels of total cholesterol (TC), LDL-C, and apoliprotein B in plasma, showing the beneficial effects of estrogen in reducing the risk of cardiovascular disease (14–21). Estrogen increases the LDL receptor (LDLR) expression to reduce the levels of total blood cholesterol and LDL-C through nuclear receptor ERα, which has been extensively investigated and is also known to be in the classical receptor pathway and to have a genomic effect (22–27). However, in C57BL/6J mice, estrogen-mediated prevention of vascular injury in response to carotid artery denudation is independent of ERα (28). Another finding suggests that protection from the development of early atherosclerotic lesions is dependent on estrogen but independent of ERα (29). Therefore, many studies strongly support a non-genomic effect of estrogen through its membrane receptor-related signal transduction (30–33).

It has been shown that the G protein-coupled estrogen receptor (GPER) mediates the rapid effect of estrogen via a non-genomic effect (31, 32, 41). The activation of the GPER by estrogen may upregulate LDLR expression and downregulate proprotein convertase subtilisin/kexin type 9 (PCSK9), a newly discovered negative regulator of lipid metabolism. As a risk factor for coronary artery disease, PCSK9 elevates LDL-C levels in plasma by binding to the extracellular domain of LDLR in hepatocytes to mediate LDLR endocytosis and degradation in hepatic lysosomes (34). In HepG2 cells expressing this receptor and the GPER agonist, G1 can decrease PCSK9 mRNA and protein levels so that LDLR protein expression increases on the cell membrane (41), thus suggesting its influence on the transcription of PCSK9. Studies in rats and humans found that high levels of endogenous estrogens reduce serum PCSK9, suggesting that estrogens also increase the number of LDLRs on hepatic cells through a posttranscriptional mechanism (35, 36). In human HuH7v hepatocarcinoma cells, LDLR mRNA is upregulated by βE2-conditioned medium with PCSK9, while βE2-conditioned medium without PCSK9 fails to upregulate LDLR expression. PCSK9-knockdown cells in medium without PCSK9 showed no increases in LDLR levels by βE2 (37). Therefore, these studies imply that PCSK9 is essential in the estrogen-induced upregulation of LDLR.

It has recently been reported that the GPER is a receptor in the plasma membrane that can be translocated to the endoplasmic reticulum (ER) (30). In HepG2 cells, βE2 is shown to be rapidly internalized via clathrin-coated vesicles and transported to sorting endosomes (38). Therefore, the aim of this study was to investigate the mechanism by which βE2 modulates PCSK9-mediated LDLR degradation and the role of clathrin in GPER activation by βE2. Our data showed that GPER activation by βE2 is dependent on phospholipase C-γ (PLCγ) and intracellular Ca^2+^ signaling, which alters the distribution of clathrin, thereby inhibiting PCSK9-mediated degradation of LDLR. These data may help understand the molecular mechanisms underlying the physiological effects of estrogens.

## Materials and Methods

### Reagents and Antibodies

Recombinant human PCSK9 (rhPCSK9, gl-10916) was purchased from Genlocus (Chengdu, China). βE2 (E2758-1G) was purchased from Sigma-Aldrich (Shanghai, China). The GPER antagonist G15 (1161002-05-6) was purchased from Cayman Chemical (Michigan, USA). The PLC inhibitor U73122 (U6756-5 MG) was purchased from Sigma-Aldrich (San Francisco, USA). Anti-LDLR antibody (sc-373830) was ordered from Santa Cruz (Dallas, USA). Anti-phospho-PLCγ (Tyr783) polyclonal antibody (2821) and anti-PLCγ rabbit antibody (2822) were purchased from Cell Signaling (Hongkong, China). Anti-clathrin rabbit polyclonal antibody (ab59710) was purchased from Abcam (Shanghai, China). The BODIPY^TM^ FL LDL (L3483), Alexa Fluor^®^ 488 protein labeling kit (A10235), Alexa Fluor^®^ 488-conjugated goat anti-mouse IgG (A11001) and Alexa Fluor^®^ 488-conjugated goat anti-rabbit IgG (A11008) were all purchased from Thermo Fisher (Shanghai, China). A cAMP parameter assay kit (KGE002B) was purchased from R&D (Minneapolis, USA). The PrimeScript™ II 1st Strand cDNA synthesis kit (6210A) and TB GreenTM Premix Ex Taq II (RR420B) were purchased from TaKaRa (Dalian, China).

### Cell Culture

HepG2 cells (ATCC, USA) were maintained at 37°C in phenol red-free DMEM supplemented with 10% fetal bovine serum, 100 IU/mL penicillin, and 100 μg/mL streptomycin and allowed to grow until 85% confluent prior to the experiment.

### PCSK9 Internalization Assay

For the internalization assay, rhPCSK9 was labeled with an Alexa Fluor^®^ 488 from the protein labeling kit (^AF−^PCSK9). To determine whether βE2 blocked the internalization of PCSK9, HepG2 cells were seeded into 48-well plates at 4 × 10^4^ cells per well in growth medium. After serum deprivation for 16 h, the cells were incubated with 25 μg/mL ^AF−^PCSK9 for 0.5–6 h and treated with 0.01–10 μM βE2 for 2 h in the presence of ^AF−^PCSK9 (25 μg/mL). For all assays, the cells were pretreated with 1 μM G15 for 15 min prior to the addition of βE2 to block GPER action. After a series of wash steps with PBS, internalized ^AF−^PCSK9 was directly observed under an inverted fluorescence microscope, and the fluorescence intensity of ^AF−^PCSK9 in isopropyl alcohol was detected by a SpectraMax M5 reader and reported in RFUs.

### LDLR Protein Assay

To investigate whether βE2 prevents PCSK9-mediated LDLR degradation, HepG2 cells were seeded into 6.2 cm^2^-cell imaging dish (NEST, Suzhou, China) at 6 × 10^4^ cells per well in growth medium and allowed to adhere to the plate for 24 h. After serum deprivation for 16 h, the cells were treated with or without 0.01–1 μM βE2 for 6 h in the presence or absence of rhPCSK9 (25 μg/mL). The expression level of LDLR was detected by western blotting and immunofluorescence.

### LDLR mRNA Assay

For detection of LDLR mRNA, HepG2 cells were seeded into 6-well plates at 1 × 10^5^ cells per well in growth medium and allowed to adhere to the plate for 24 h. After serum deprivation for 16 h, the cells were incubated with or without 0.01–1 μM βE2 for 6 h in the presence of rhPCSK9 (25 μg/mL). Total RNA was extracted using TRIzol reagent (Invitrogen) according to the manufacturer's protocol. cDNA was prepared from 1 μg RNA using a PrimeScript™ II 1st strand cDNA synthesis kit according to the manufacturer's protocol (TaKaRa). Real-time quantitative PCR was performed to detect the expression of LDLR mRNA in a CFX96 PCR system (Bio-Rad) with 1 μL of cDNA diluted 25-fold in 12.5 μL of the reaction mixture with TB Green Premix Ex Taq II and 0.1 μM forward and reverse primers for LDLR and β-actin. Specific primers for LDLR and β-actin were used: for LDLR, forward, 5′-GTTATTCAGGGAGAACGGCT-3′ and reversse, 5′-GAGTCAACCCAGTAGAGGC-3′; and for β-actin, forward, 5′-CTGGGCATGGAGTCCTGTG-3′, and reverse, 5′-ATCTTCATTGTGCTGGGTG-3′. The primers were obtained from GENEWIZ (Suzhou, China). We set amplification parameters: 95°C for 30 s; 40 cycles of 95°C for 5 s; and 56°C for 30 s.

### LDL Uptake Assay

LDL uptake was measured using a BODIPY^®^ FL kit. HepG2 cells were seeded into 48-well plates at 4 × 10^4^ cells per well in growth medium and allowed to adhere to the plate for 24 h. After serum deprivation for 16 h, the cells were treated with or without 0.01–10 μM βE2 for 4 h in the presence of rhPCSK9 (25 μg/mL). For the assay, the cells were pretreated with 1 μM G15 for 15 min prior to the addition of βE2 to block GPER action. BODIPY^TM^ FL LDL (6 μg/ml) was added for 3 h, and then the cells were then washed with PBS. Isopropyl alcohol was added to measure the uptake of BODIPY^TM^ FL LDL. The plates were read with a SpectraMax M5 reader (Molecular Devices, Sunnyvale, USA).

### Clathrin Assay

Clathrin was also measured for the distribution assay. HepG2 cells were seeded into 6.2 cm^2^-cell imaging dish (NEST, Suzhou, China) at 6 × 10^4^ cells per well in growth medium and allowed to adhere to the plate for 24 h. After serum deprivation for 16 h, the cells were incubated with 25 μg/mL rhPCSK9 for 0–2 h and treated with 0.01–1 μM βE2 for 2 h in the presence of rhPCSK9 (25 μg/mL). For all assays, the cells were pretreated with 1 μM G15 for 15 min prior to the addition of βE2 to block GPER action. After a series of wash steps with PBS, clathrin was detected by immunofluorescence with the corresponding antibodies.

### Calcium Mobilization Assay

Calcium mobilization was detected by Fluo-4 AM (Invitrogen, Shanghai, China). HepG2 cells were seeded in a cell imaging dish at 6 × 10^4^ cells per well and allowed to adhere to the plate for 24 h. After serum deprivation for 16 h, the cells were washed with Ca^2+^-free HBSS (136.8 mM NaCl, 5.4 mM KCl, 5.5 mM Glucose, 0.44 mM KH_2_PO_4_, and 0.33 mM Na_2_HPO_4_, pH 7.2) and then loaded with 5 μM Fluo-4 AM for 20 min. The cells were washed again with Ca^2+^-free HBSS. Each cell imaging dish was placed in a laser scanning confocal microscope (LSM710, ZEISS). First, the fluorescence intensity images were collected every 15 s for 5 min, and 0.1 μM E2 was then added and fluorescence was monitored continuously for 15 min. For the assay, the cells were treated with 1 μM G15 and 1 μM U73122 for 15 min prior to the addition of E2 to block GPER and PLCγ action. Data were quantified and analyzed using ZEN 2010 software. The background was subtracted, and the fluorescence was expressed relative to values at the beginning of the experiment.

### Western Blot Analysis

HepG2 cells were seeded into T25-cell culture bottles at 5 × 10^5^ cells with 5 mL growth medium. After serum deprivation for 16 h, the cells were treated with or without 0.1 μM βE2 for 5 min in the presence of rhPCSK9 (25 μg/mL). For the assay, the cells were pretreated with 1 μM G15 for 15 min prior to the addition of βE2 to block GPER action. After treated with 0.1 μM βE2 for 5 min or 0.01–1 μM βE2 for 6 h, the cells were washed twice with cold PBS and lysed with 1 × RIPA buffer (Beyotime, China) supplemented with a protease inhibitor PMSF (1 mM) to obtain the total protein. An equal amount of total protein from each lysate was loaded and separated on 4%−10% gels for SDS-PAGE and electroblotted to polyvinylidene fluoride (PVDF) membranes. The primary antibodies used included anti-LDLR mouse monoclonal antibody (1:150 diluted in PBS), anti-pPLCγ (Tyr783) rabbit antibody (1:500 diluted in PBS), and anti-PLCγ rabbit antibody (1:500 diluted in PBS). The secondary HRP-conjugated antibody was goat anti-mouse IgG (1:4000 diluted in PBS) and goat anti-rabbit IgG (1:4000 diluted in PBS). Western blots were measured for chemiluminescence (Thermo Fisher), detected by ImageQuant LAS 500 (General Electric Company, Boston, USA), and analyzed by Image Plus 6.0 analysis software.

### Immunofluorescence Staining

Experiments were performed in cell imaging dishes as described in the methods section LDLR Protein Assay and Clathrin Assay. HepG2 cells were washed three times with PBS and fixed with a 4% paraformaldehyde solution for 20 min. The fixed cells were permeabilized with PBS containing 0.3% Triton X-100 for 3 min at 4°C and washed three times with PBS containing 0.5% BSA. The cells were incubated with 3% BSA in PBS for 30 min at 37°C and then incubated with primary antibodies: anti-LDLR mouse monoclonal antibody (1:100 diluted in PBS) and anti-clathrin rabbit polyclonal antibody (1:500 diluted in PBS) at 4°C overnight. Antibodies were stained with Alexa Fluor^®^ 488-conjugated goat anti-mouse IgG (1:250 diluted in PBS) and Fluor^®^ 488-conjugated goat anti-rabbit IgG (1:250 diluted in PBS) at room temperature for 1 h. Excess antibody was removed by washing the cells with PBS, and the cells were stained with 4′6-diamidino-2-phenylindole (DAPI) and then viewed with a confocal microscope (LSM710, Carl Zeiss AG, German).

### Statistical Analysis

Data are presented as the mean ± SEM. Differences among groups were tested by SPSS 17.0 software with one-way ANOVAs followed by an LSD or Duncan test. Comparisons were considered to be statistically significant if *P* < 0.05.

## Results

### β-Estradiol Blocked PCSK9 Internalization

**Figure 1:**
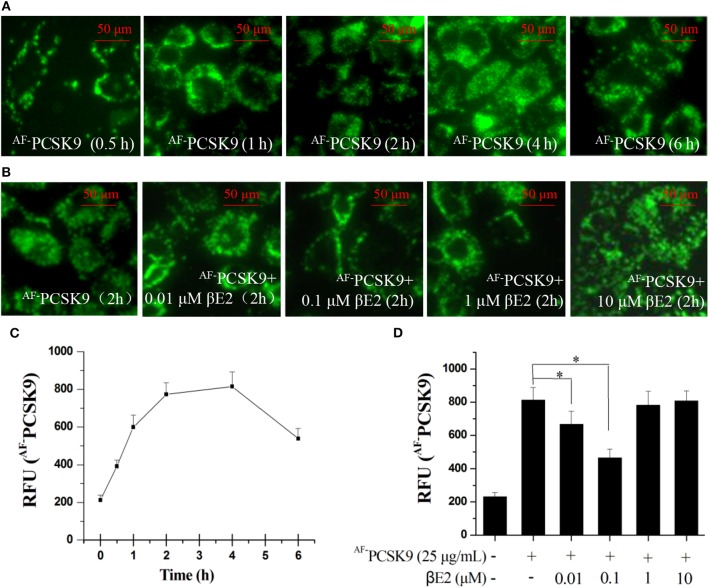
βE2 blocked the internalization of PCSK9. **(A)** Alexa Fluor 488 dye-labeled PCSK9 (25 μg/mL) was added to HepG2 cells not treated with βE2. ^AF−^PCSK9 internalization was observed at specified time intervals. **(B)** Internalization of ^AF−^PCSK9 was blocked at the cell surface by βE2 administered at specified concentration ranges. **(C,D)** Quantification of ^AF−^PCSK9. The relative fluorescent unit (RFU) of ^AF−^PCSK9 was determined by ZEISS 2010 software. Values represent the means ± SEM, *n* = 3; *P < 0.05 for a comparison between two groups.

PCSK9 is a secreted protein and negative regulator of LDLR in hepatocytes. It binds to LDLR at the cell surface and can mediate LDLR into lysosomes, where it is degraded. We first examined PCSK9 internalization using an Alexa Fluor 488 dye-labeled rhPCSK9 (^AF−^PCSK9) in HepG2 cells expressing LDLR. The fluorescence signals were detectable in the HepG2 cells at 30 min and peaked at 2 and 4 h after ^AF−^PCSK9 (25 μg/mL) was added to the cells. The localization of ^AF−^PCSK9 was initially at the cell surface, and it was later localized throughout the cytoplasm (Figure 1A) with increasing quantity (Figure 1C), indicating the internalization and intracellular trafficking of ^AF−^PCAK9.

To determine whether βE2 blocked the internalization of PCSK9, HepG2 cells were incubated with 0.01–10 μM βE2 for 2 h in the presence of ^AF−^PCSK9. In the HepG2 cells without βE2, the fluorescence signals were distributed throughout the cytoplasm after ^AF−^PCSK9 (25 μg/mL) was added. In contrast, βE2 treatment decreased the cytoplasmic distribution of ^AF−^PCSK9 and increased the distribution of ^AF−^PCSK9 at the cell surface (Figure 1B), and this effect was most pronounced with the βE2 treatment of 0.1 μM (Figure 1D). These results indicate that estrogen can block the internalization of PCSK9 and thus may prevent PCSK9-mediated LDLR degradation. In cultured HepG2 cells, a high concentration of βE2 (10 μM) led to significant cytotoxicity. Therefore, all subsequent experiments were performed with 0.01–1 μM βE2 treatments.

### βE2 Prevented PCSK9-Mediated LDLR Degradation

**Figure 2:**
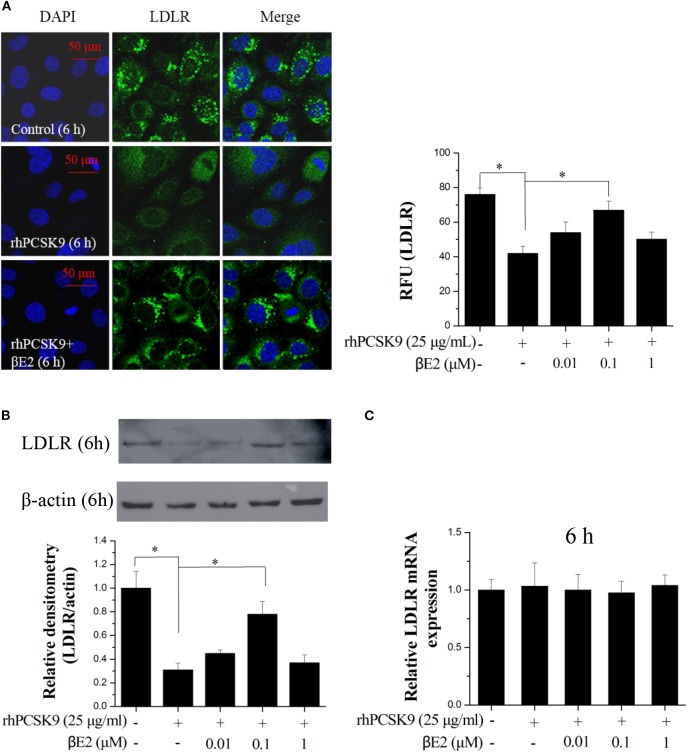
βE2 treatment prevented PCSK9-mediated LDLR degradation in HepG2 cells. **(A)** βE2 treatment increases the fluorescence intensity of LDLR, as determine by immunofluorescence assay. The relative fluorescent unit (RFU) of LDLR was determined by ZEISS 2010 software. **(B)** βE2 treatment increased LDLR protein levels in the presence of rhPCSK9 (25 μg/mL) by Western blot analysis. **(C)** qPCR was used to examine the mRNA levels of LDLR in the HepG2 cells treated with βE2 for 6 h in the presence of rhPCSK9 (25 μg/mL). The β-actin value was used to normalize the qPCR results. Values represent the means ± SEM, *n* = 3; *P < 0.05 for a comparison between two groups.

As expected, the immunofluorescence from the stained LDLR was significantly enhanced after treatment with βE2 at 0.1 μM but not 1 μM for 6 h compared with that in the control HepG2 cells that were treated only with PCSK9 (Figure 2A). A similar result was obtained using Western blotting (Figure 2B). βE2 treatment at 0.1 μM significantly increased LDLR levels in the presence of rhPCSK9 (25 μg/mL), and βE2 treatment at 1 μM did not increase LDLR levels. In addition, LDLR mRNA levels were detected by qPCR, and no change was observed following βE2 treatment (Figure 2C). The addition of rhPCSK9 to the HepG2 cells for 6 h resulted in the reduction in LDLR levels, indicating that PCSK9 mediated LDLR degradation. These data suggest that βE2 treatment at 0.1 μM can inhibit PCSK9-mediated LDLR degradation in HepG2 cells.

### βE2 Modulates Cellular LDL Uptake in the Presence of PCSK9

**Figure 3:**
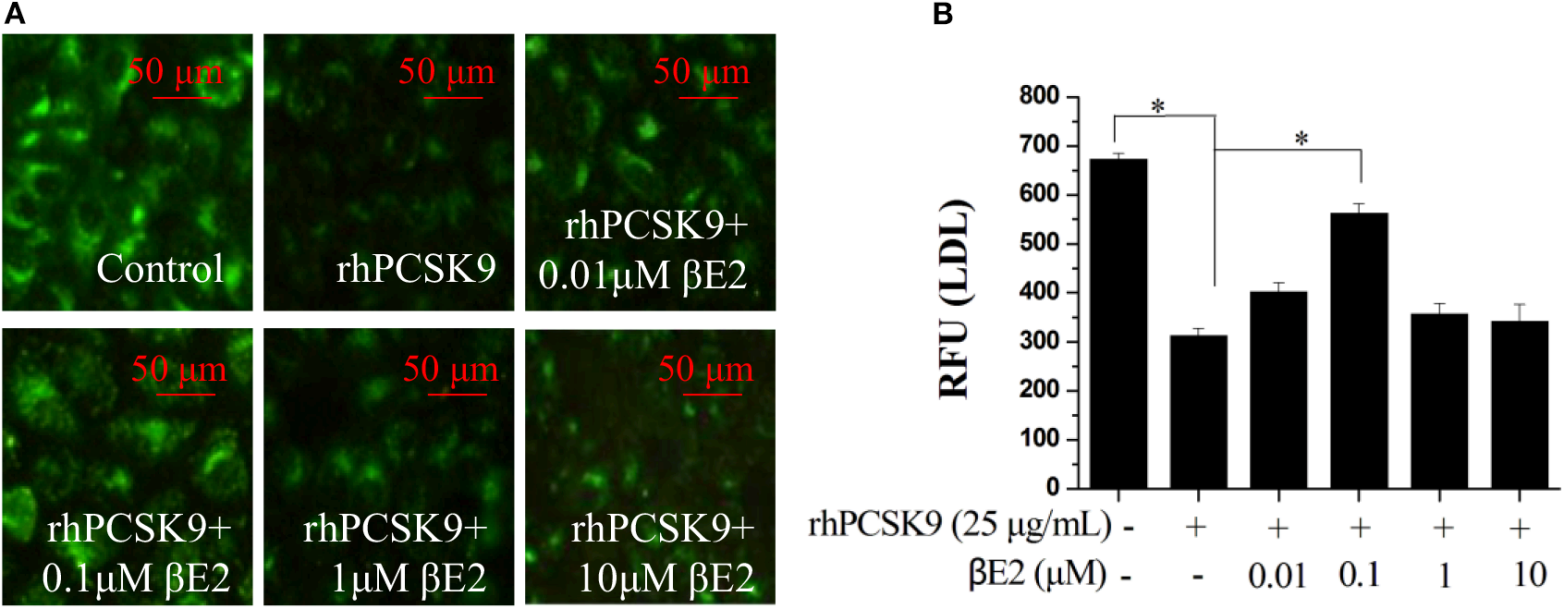
βE2 treatment for 4 h promoted LDL uptake in the presence of rhPCSK9 (25 μg/mL). **(A)** LDL uptake was visualized in the HepG2 cells using BODIPY-labeled LDL and detected by fluorescence microscopy as shown in the representative images. **(B)** BODIPY^®^ FL fluorescence was quantified with a SpectraMax M5 reader. Values represent the means ± SEM, *n* = 3; *P < 0.05 for a comparison between two groups.

The addition of recombinant PCSK9 resulted in LDLR degradation. The inhibitory effect of βE2 on PCSK9-mediated LDLR degradation may promote the uptake of LDL-C into cells. As shown in Figure 3, βE2 treatment increased the fluorescence intensity of the labeled LDL in the HepG2 cells compared to the intensity observed with rhPCSK9 treatment alone, indicating that βE2 promotes LDL uptake into cells in the presence of rhPCSK9. Consistent with the levels of LDLR protein expression, βE2 treatment at 1 μM did not promote LDL uptake.

### βE2 Prevents PCSK9-Induced Increases in Clathrin

**Figure 4:**
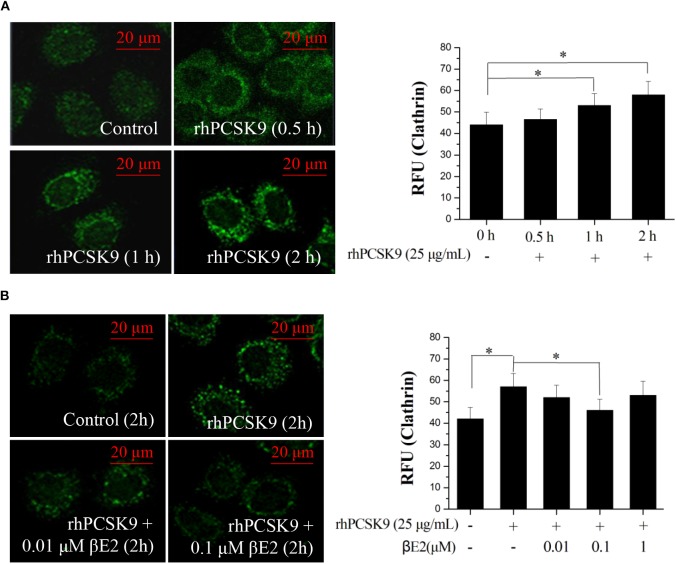
βE2 treatment prevented the PCSK9-induced recruitment of clathrin. **(A)** HepG2 cells were incubated in the presence of rhPCSK9 (25 μg/mL), and clathrin levels were measured the specified time intervals. **(B)** HepG2 cells were treated with βE2 (0.01–1 μM) for 2 h in the presence of rhPCSK9 and were visualized for the detection of immunofluorescence-stained clathrin. The fluorescent quantification of clathrin was determined by ZEISS 2010 software. Values represent the means ± SEM, *n* = 3; *P < 0.05 for a comparison between two groups.

Previous studies have suggested that clathrin trafficking is involved in PCSK9-mediated LDLR degradation in hepatic cells. The major route of PCSK9-dependent LDLR degradation in HepG2 cells has been demonstrated to be through clathrin-mediated endocytosis (39). Knocking down the clathrin heavy chain increased the LDLR protein levels in HuH7 cells (40), indicating that clathrin is involved in PCSK9-mediated LDLR degradation in hepatocytes. We asked whether clathrin trafficking pathway might also be affected by βE2. HepG2 cells were incubated with rhPCSK9 for 0–2 h and then examined for clathrin distribution by staining an antibody to clathrin. The addition of rhPCSK9 caused an increase in clathrin stain intensity in HepG2 cells, with large cytoplasmic and perinuclear localization peaks at 2 h, in contrast to the weak staining observed in the absence of rhPCSK9 (Figure 4A). In parallel experiments, clathrin staining was decreased by βE2 treatment (Figure 4B). These results suggest that PCSK9 may induce an increase in clathrin levels. It is very likely that clathrin is recruited by the addition of rhPCSK9 and then participates in PCSK9-mediated LDLR degradation in HepG2 cells. βE2 may prevent clathrin-dependent LDLR endocytosis, thereby inhibiting PCSK9-mediated LDLR degradation.

### βE2 Prevents PCSK9-Mediated LDLR Degradation by Activating the GPER

**Figure 5:**
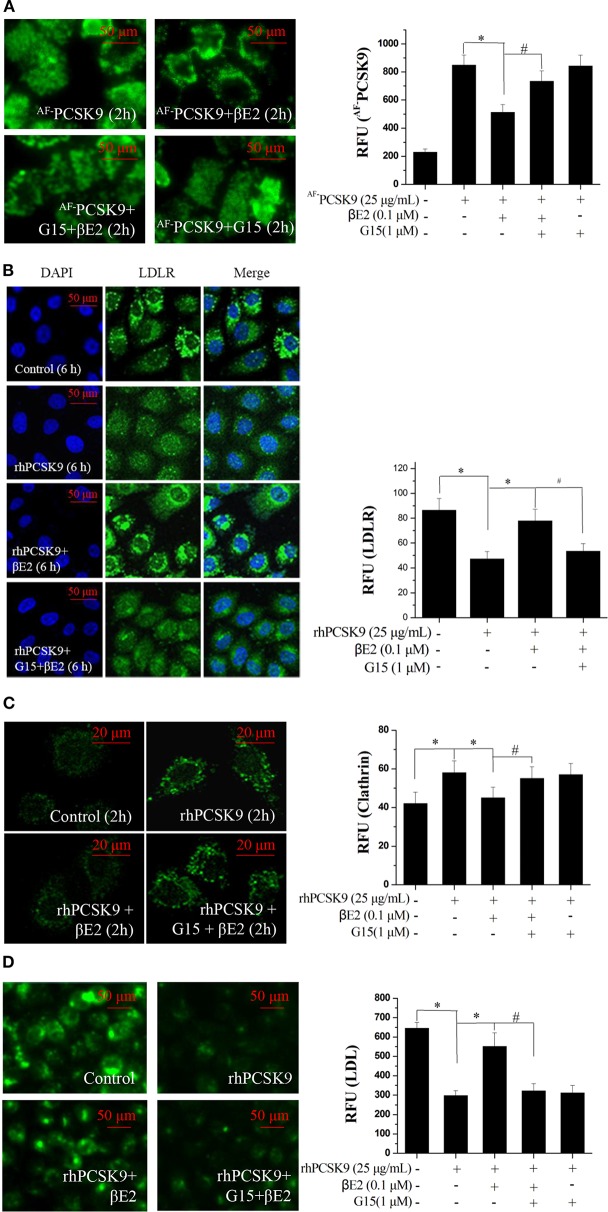
βE2 treatment prevented the PCSK9-mediated LDLR degradation by activating GPR30. **(A)** βE2-treated cells for 4 h with and without G15 were visualized for the internalization of ^AF−^PCSK9 (25 μg/mL) by fluorescence microscopy. **(B,C)** Immunofluorescence staining of LDLR and clathrin, and the intensity of which was quantified in βE2-treated cells treated with and without G15, respectively. **(D)** After 4 h, the cells were exposed to BODIPY-labeled LDL, and then, the uptake was visualized through fluorescence microscopy. Fluorescence intensity was quantified with a SpectraMax M5 reader. Values represent the means ± SEM, *n* = 3, *P < 0.05 and ^#^*P* < 0.05 for comparisons between two groups.

The GPER may function uniquely as an intracellular transmembrane receptor to contribute to normal estrogen physiology as well as pathophysiology (30). In particular, GPER activation has been shown to upregulate LDLR expression and consequently LDL metabolism (41). To determine whether the effect of βE2 was mediated through the GPER, HepG2 cells were pretreated with G15, a selective GPER antagonist, followed by 0.1 μM βE2 treatment for 2 h in the presence of ^AF−^PCSK9. As shown in Figure 5A, ^AF−^PCSK9 was mainly distributed at the cell surface after 0.1 μM βE2 treatment for 2 h. However, G15 pretreatment for 15 min increased the distribution of ^AF−^PCSK9 in the cytoplasm, exhibiting a similar distribution as that of the ^AF−^PCSK9 to control cells not treated with βE2. G15 pretreatment also inhibited the effects of βE2, increasing LDLR degradation (Figure 5B) and clathrin levels (Figure 5C). In addition, βE2-enhanced LDL uptake was prevented by G15 pretreatment (Figure 5D).

### βE2 Promotes Rapid Intracellular PLC Signaling Through GPER Activation

**Figure 6:**
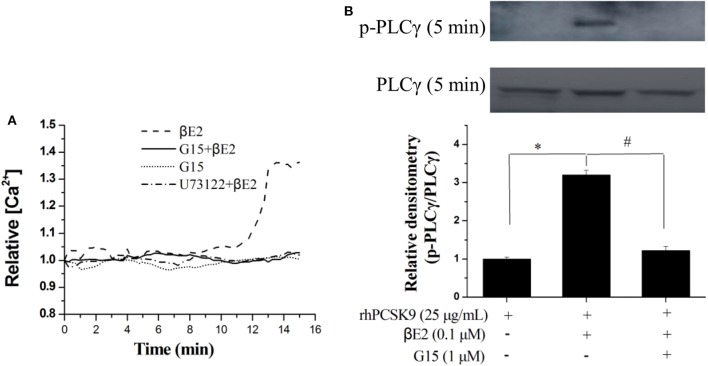
βE2 treatment promoted rapid intracellular PLC signaling through GPER activation. **(A)** βE2 treatment promoted the GPER-dependent release of intracellular calcium. **(B)** βE2 treatment increased p-PLCγ protein levels through GPER activation, as determined by Western blot analysis. Values represent the means ± SEM, *n* = 3; *P < 0.05 and ^#^*P* < 0.05 for a comparison between two groups.

The GPER is involved in intracellular calcium mobilization in different cell types, and its role is related to the rapid activation of phospholipase C. We next investigated the rapid effect of βE2 on GPER activation by measuring the release of intracellular calcium. Intracellular Ca^2+^ was released from in HepG2 cells after 15 min of 0.1 μM βE2. The effects of βE2 can be blocked by G15 and U73122 (PLC inhibitor) pretreatment of cells (Figure 6A) for 15 min. Phospholipase C-γ (PLCγ), in the Ca^2+^ signaling pathway, was also activated after a 5 min βE2 incubation. The effect of βE2 can be blocked by G15 pretreatment (Figure 6B), indicating the rapid activation of PLCγ through GPER stimulation.

## Discussion

Several studies have been reported to suggest that estrogen regulates lipoprotein metabolism through the GPER in human hepatic HepG2 cells or in HuH7 cells (37). However, no detectable change in PCSK9 transcription levels was found with increased βE2 doses (37). βE2 may upregulate LDLR through extracellular PCSK9-dependent mechanisms in human hepatic cells (37). The results presented here provide data in support of this hypothesis, i.e., that the effect of βE2 regulation of LDLR is dependent upon extracellular PCSK9. There was a significant increase in LDLR protein expression upon βE2 treatment in the presence of rhPCSK9. Moreover, βE2 inhibits PCSK9-mediated LDLR degradation, which results in the increase of LDL uptake by HepG2 cells, an effect that was blocked by G15, a GPER antagonist.

It has been shown that PCSK9 binds to the extracellular domain of LDLR to trigger LDLR degradation in lysosomes (39, 42, 43). Clathrin is a cytosolic protein, and the importance of the clathrin heavy chain has been demonstrated through the PCSK9-dependent degradation of LDLR (39). Although there is no evidence that clathrin recruitment is affected by PCSK9, the HepG2 cells treated with rhPCSK9 had increased levels of clathrin in the cytoplasm and perinucleus. It is likely that PCSK9 rapidly binds to LDLR to form a PCSK9-LDLR complex that recruits clathrin, and the complex then enters cells by a clathrin-dependent endocytosis pathway that leads to LDLR degradation. βE2 may prevent clathrin-dependent LDLR endocytosis, thereby inhibiting PCSK9-mediated LDLR degradation, which was blocked by G15. Therefore, βE2 increases LDLR protein expression, likely via GPER activation.

GPER also contributes to the activation of the phospholipase C (PLC) pathway in other cell types (44). The activation of PLC may lead to an increase in intracellular Ca^2+^ and regulated clathrin-dependent endocytosis (45, 46). In HepG2 cells, PLC-gamma (PLCγ) was activated, and intracellular Ca^2+^ release was detected after βE2 treatment. These effects of βE2 were blocked by the GPER antagonist G15 and the PLC inhibitor U73122, indicating a role for estrogen in the rapid regulation of LDLR lysosomal degradation through GPER. The regulation of PCSK9 levels has been shown to be cAMP-dependent (47), and cAMP is another messenger pathway known to be regulated by GPER activation. We also demonstrate that βE2 rapidly stimulates cAMP production which was blocked by G15 (data not shown).

**Figure 7:**
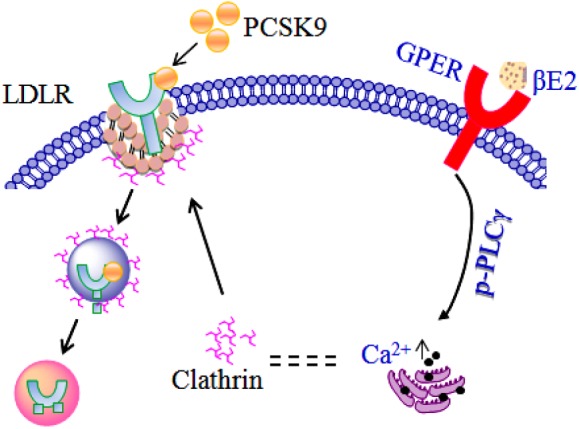
Model of the regulation of LDLR by estrogen in the presence of PCSK9.

Figure 7 presents a schematic diagram summarizing our results. PCSK9 binds to the extracellular domain of LDLR to mediate LDLR endocytosis and degradation in a clathrin-dependent manner. The molecular mechanism of estrogen inhibition of PCSK9-mediated LDLR degradation can be explained: activating GPER in the membrane by βE2 (48) rapidly initiates the phosphorylation of PLCγ, leading to intracellular Ca^2+^ release, which in turn alters clathrin distribution or influences the clathrin trafficking pathway to affect the internalization of the PCSK9-LDLR complex. Another possible explanation is that the GPER is localized to the endoplasmic reticulum (30), where the binding of βE2 initiates activation of PLCγ and recruits calthrin assembly proteins to it, forming a clathrin-coated pit (38). The coated pit is internalized and prevents clathrin-dependent LDLR endocytosis, thereby inhibiting PCSK9-mediated LDLR degradation. The GPER may function uniquely as an intracellular transmembrane receptor that contributes to normal estrogen physiology as well as pathophysiology (30). Investigations into other possible intracellular GPER isoforms activated by estrogen would be of great interest.

In summary, our results highlight the important role of estrogen in regulating LDLR levels through GPER activation and being associated with clathrin trafficking pathway. Therefore, estrogen may have a role in modulating the levels of total blood cholesterol and LDL-C in hyperchlesterolemic women.

## Data Availability Statement

All datasets generated for this study are included in the article/supplementary material.

## Author Contributions

WF designed and planned the study and performed the experiments. X-PG and L-MY designed and planned the study and wrote the manuscript. SZ, Y-PD, and W-JZ performed the experiments and analyzed the data.

### Conflict of Interest

The authors declare that the research was conducted in the absence of any commercial or financial relationships that could be construed as a potential conflict of interest.
